# Supplementary material for: Are carbon emissions trading and green financial instruments synergistic? -Comprehensive quantitative research based on content analysis
Source: PLoS One. 2024 Mar 7;19(3):e0298601. doi: 10.1371/journal.pone.0298601 (PMC10919601; doi:10.1371/journal.pone.0298601)
Supplement: S1 Data — (ZIP) [file pone.0298601.s001.zip › Data/地级市Z_F工作报告.zip!/║┌┴·╜¡/║┌║╙╩╨/└┤╫╘╣1⁄2╓┌║┼í╛┬φ┐╦╩2╛▌═°í┐_2011.docx]

各位代表：

现在，我代表市人民政府向大会作工作报告，请审议，并请列席会议的同志提出意见。

一、“十一五”及2010年工作回顾

“十一五”是我市发展进程中极不平凡的时期。在省委、省政府和市委的正确领导下，在市人大、政协的监督支持下，我们紧紧依靠全市人民，深入贯彻落实科学发展观，积极应对金融危机影响和宏观形势变化，在各种困难和风险中开拓前进，经济社会发展取得了重大成就。城镇固定资产投资提前一年，地区生产总值、人均地区生产总值提前两年，全口径财政收入、工业增加值提前三年，对外贸易进出口总值提前四年完成“十一五”规划目标。五年来，坚持发展第一要务，经济整体实力显著提升。始终把发展牢牢抓在手上，凝神聚力、埋头苦干，全市经济呈现总量扩张、增速加快、效益提升的良好态势。农业生产连年迈出新步伐，深入实施“双增”工程，粮食产量大幅提升，比“十五”末增长54.2%，122个新农村建设试点村通过验收，农业发展基础更加稳固。工业经济强筋健骨，全市规模以上工业增加值比“十五”末增长3.7倍，年均增长28%。地方规模以上工业利税比“十五”末增长5.3倍，年均增长39%。省级以上园区发展到5个，入区企业44户。以“四型经济”为引领的第三产业较快发展，景区景点建设进一步加快，一系列重大活动成功举办，影响力持续提升，人气商气日益旺盛。累计接待旅游者886万人次，是“十五”时期的1.5倍，年均增长27.1%。社会消费品零售总额年均增长15.8%，高于预期目标5.8个百分点。坚持项目引领发展，支撑保障能力明显增强。立足自身优势，把发展目标细化落实到每一个具体项目上，强力推动投资增长。五年实施重点产业项目50项，总投资405亿元，超亿元、10亿元和20亿元的分别为37项、9项和4项。全市城镇固定资产投资比“十五”末增长4倍，占GDP比重从2005年的22.3%提高到54.1%。通过依托企业、展会、驻外工作组等多种招商方式，落实引资项目913项，到位资金239.4亿元。累计有15个项目列入省财源项目库。加强了煤炭、建安等重点领域税收征管，仅煤炭企业税收就实现7亿元。全市全口径财政收入于2008年和2010年先后突破10亿元和20亿元大关。坚持推进改革开放，区域发展活力不断激发。不断探索符合黑河实际的新思路、新举措，努力破解制约发展的难题，加快创新步伐，构建大开放格局。国有林权改革试点在全省率先启动，集体林权改革基本完成。适应现代农业发展需要，加快推进土地规模经营，流转面积占全市土地承包面积的48%。探索实施了“五化”新机制，城市管理更加规范。森防“两个机制、五个系统”逐步完善，连续9个防期没有发生重特大火灾，实现历史性突破。政府机构、财政、投融资、医疗等方面改革也扎实推进。面向国际国内两个市场，全面推进对外开放，五年对外贸易进出口总值实现115.2亿美元，对俄投资2.4亿美元，合作项目20项，吸引外资2.1亿美元。坚持改善民生条件，群众生活质量持续提高。落实科教兴市和人才强市战略，累计实施科技项目181项，引进储备重点院校和急需专业大学毕业生489名。以创业带动就业，城镇五年新增就业8万人，城镇登记失业率始终保持在4%以内较低水平。优化教育资源配置，投资6.9亿元，新建维修中小学校舍253所33.8万平方米，整合183所，顺利通过国家“两基”验收。市县两级新建改造医院、疾控中心8所和乡镇卫生院61所，城乡医疗卫生条件不断改善。城镇医疗保险覆盖率88%，新农合参合率99.6%。稳定低生育水平，生育符合政策率始终保持在97%左右。公共文化体系日益完善，群众文化活动丰富多彩，城乡举办各类演出5100余场次。竞技体育水平不断提高，群众性体育运动蓬勃发展，共获得省级以上奖牌494枚，体育人口达到40万人。城镇居民最低生活保障标准由每月150元提高到251元，实现了应保尽保。多渠道增加群众收入，改善生产生活条件。全市城镇居民人均可支配收入和农村居民人均纯收入分别增长1.4倍和1.1倍。城市新增建筑面积239万平方米，城镇和农村人均住房建筑面积分别达到26.2平方米和19.7平方米。新增供热面积999.7万平方米，其中市区280万平方米。各项基础设施建设向农村延伸，基本实现了村村通电话、通广播电视。全市私家车保有量4.67万辆，每100户拥有电脑25台。坚持打牢发展基础，经济保障环境更加优化。把更多精力放在培育和优化环境上，为经济社会发展提供强劲保障。打造舒适的生活环境，在全省率先编制了城市风貌规划，滨水城市建设步伐明显加快，五年累计投入基础设施建设资金24.8亿元，是“十五”时期的4倍，“三优”文明城市创建工程走在全省前列。打造良好的生态环境，全面停止国有林木商业性采伐，累计植树造林110.9万亩，林业产业产值增长4倍。我市整体纳入大小兴安岭生态保护与经济转型规划，节能减排、污染治理等工程取得积极进展，建成黑河、北安、嫩江、五大连池污水处理厂，万元地区生产总值能耗比“十五”末下降18%。打造便捷的交通环境，新建改造公路7359公里，乡镇和行政村通畅率分别达到100%和98%。打造良好的稳定环境，安全生产形势逐年好转，信访调处机制不断健全，应急体系初步建立，治安防控效果明显，群众安全感进一步增强。打造高效的政务环境，推进了电子政务和政务公开，行政审批事项由191项精简到115项，出台政府重大决策议事规则和各类管理办法40个。实行了入企报告制和责任追究制，吸引力和竞争力明显增强，相继荣获“中国魅力中小城市”、“最具投资潜力城市二十强”等荣誉称号。

各位代表，2010年是“十一五”收官之年。我们以产业发育为主线，以项目建设为支撑，经过不懈努力，圆满完成了市四届人大四次会议确定的发展目标和重点任务。预计全市生产总值256亿元，同比增长14%。全社会固定资产投资142.9亿元，增长45.9%。社会消费品零售总额54.3亿元，增长18.3%。全口径财政收入24.6亿元，增长42.3%。城镇居民人均可支配收入1.2万元，农村居民人均纯收入7045元，分别增长20%和25.6%。万元地区生产总值能耗下降4.35%。一年来，主要做了以下工作：

——把重点项目建设作为经济工作的重中之重，着力促进投资持续快速增长。全市新建续建重点项目56项，完成投资125亿元，增长106%，创历史最高水平。合作区多晶硅、爱辉林板一体化、北安汉麻纺织等7个市长包保推进项目全部开复工，其中风景区矿泉水项目完工。黑河新兴墙体材料、北安庆华机械装备制造、昌泰水泥和嫩江嵩天薯业一期等项目投产。五大连池矿泉啤酒、逊克东安岩金矿、孙吴辰星马铃薯加工等项目开工。京加高速公路嫩江段交工通车，绥北高速公路、吉黑高速公路北黑段、加黑公路罕达汽至黑河段完成部分路面工程，嫩黑公路、前嫩公路、吉黑高速辅道开工，北五铁路完成可研，五大连池机场前期进展顺利。落实招商引资项目227项，到位资金76.7亿元，增长47.5%，其中驻外招商组洽谈项目63项，落地开工20项。

——把发展现代农业作为增产增收的基本途径，着力巩固农业基础地位。农业增加值62.7亿元，增长14%。粮食总产263.4万吨，增长20.6%，创历史最高水平。绿色有机农产品面积808万亩。创建高产田872.4万亩，实施标准化栽培1526万亩。流转土地460万亩，适度规模经营749万亩，分别增长25%和23%。林业产业产值18亿元，增长10%。牧业增加值8.1亿元，增长10%。渔业产值8950万元，增长17.3%。转移农村劳动力18万人，实现劳务收入11.2亿元。黑河月亮湾护岸、孙吴二门山引水、逊克奇克护岸、爱辉卧牛湖水库除险加固等工程完工，新建灌溉工程288处、饮水安全工程76处。爱辉燕麦加工、嫩江生猪加工等项目开工，龙头企业51户，实现产值33亿元，分别增长21%和22%。投入资金6.9亿元，新建大型农机合作社47个，更新改造55个。农村专业合作组织638个，增长113%。新建农村公路1407公里。局市共建领域不断拓展，与省农垦总局签订了合作共建框架协议。合作组建现代农机合作社8个。完成农机代耕、代种、代收面积600万亩，增长84%。共建农产品生产基地650万亩。

——把发育新兴产业作为提质增效的主攻方向，着力提高工业经济整体素质。全市规模以上工业增加值22.5亿元，增长27.5%。地方规模以上工业利税7.2亿元，增长30%。“三新”产业初具规模，合作区聚乙烯醇及配套电石化工项目完成投资8亿元，维生素C和聚乙烯管材、嫩江北药加工等项目开工。爱辉HPV疫苗产业化项目列入省战略性新兴产业“十二五”规划，太阳能并网发电、瑞替普酶冻干制剂等项目完成前期。逊克生物质秸秆固化项目试生产。辟建了庆华新能源战略产业园。节能减排得到加强，8家国家重点监控企业污染源监控设备投入运行，黑河粉煤灰和嫩江煤矸石综合利用等项目列入省资源节约和环境保护备选项目。

——把贸易投资互动作为恢复增长的积极举措，着力提升对外开放效益。对外贸易进出口总值28.3亿美元，增长50%。口岸出入境104.5万人次，增长21.6%。口岸货运量22.9万吨，增长10%。地产品出口2468万美元，增长3.3倍。黑河口岸首次进口煤炭5.5万吨。经营主体进一步壮大，有业绩企业214家，其中超亿美元6家。完成境外森林采伐1.6万立方米，土地开发90万亩，工程建筑装饰12.5万平方米。对俄果蔬出口基地面积达到8万亩。境外汽车4S店正式营业，玻璃深加工项目建成投产，建筑装饰材料综合市场开始装修，水泥粉磨站项目完成前期。合作区阿穆尔—黑河油品储运项目进入核准阶段。黑河港内港改造全部完成，浮箱固冰通道启动设计。

——把壮大“四型经济”作为扩大消费的重要支撑，着力增强对经济增长拉动作用。接待旅游者267万人次，增长23.4%，其中国内旅游者233万人次，边境游34万人次，分别增长21.8%和35.6%，实现旅游收入39.8亿元。黑河被评为“中国优秀旅游城市”，出境旅游实现在黑河做照。五大连池景区申遗进入国际评估审核程序，旅游名镇启动建设。五大连池景区被评为5A级旅游区，爱辉历史陈列馆、孙吴胜山要塞和卧牛湖风景区、五大连池青山公园分别被评为4A和3A级旅游区。爱辉镇旅游总体规划编制完成。黑河—布市—布列亚河旅游线路开通。试车企业和研发机构达到73家，收入比年初目标增长15%。大黑河岛国际经贸洽谈会等重大活动成功举办，组织和承办各级各类会议507次，聚集人员2.6万余人次。俄方持简化手续入境人员27.5万人次，增长40.3%。批发业、零售业、住宿业和餐饮业零售额分别增长19%、19%、25.6%和17.8%。“万村千乡”市场工程339个农家店全部通过验收。

——把培植财源作为壮大财政实力的关键环节，着力提升财政金融保障水平。全市一般预算收入15亿元，增长52.4%。一般预算支出93.1亿元，增长61.4%。市直全口径财政收入7.5亿元，增长31.6%，一般预算收入3.7亿元，增长34.3%。一般预算支出15.1亿元，增长38.7%。爱辉、北安、嫩江、五大连池、孙吴、逊克和风景区一般预算收入分别完成1.7亿元、2亿元、4.8亿元、0.78亿元、0.4亿元、1.3亿元和0.24亿元，分别增长62.3%、42.1%、88%、58.7%、60%、18.6%和28.5%。煤炭行业完成税收3亿元，增长43%。市直纳入预算管理的非税收入4亿元，增长55.6%。全市向上争取资金54.8亿元，增长23.7%，其中市直8.9亿元，增长25.6%。电石化工、多晶硅等6个项目列入省级重点财源项目。金融机构各项存款余额380.7亿元，各项贷款余额176亿元，比年初分别增长13.8%和26.2%。

——把建设宜居宜业宜乐幸福城市作为工作目标，着力推动文明城市创建工程。全市城建基础设施投入6.67亿元，其中市区2.2亿元。北安、嫩江、五大连池、孙吴和逊克分别投入1.63亿元、1.75亿元、0.57亿元、0.22亿元和0.3亿元。市区新建道路5条，沥青罩面6条，完成中央街东段步行道和黑龙江堤顶中段改造、西部棚户区5条街路步行道铺装、兴安街人防建设等工程。龙科街和铁路街等重点区域绿化，王肃街、通江路、铁路街绿篱景观亮化工程完工。大黑河岛交通桥、步行栈道桥、西部天然生态植物园及江天广场投入使用。立面改造装饰楼体65栋，亮化建筑115栋。新增城市绿地63.3万平方米，黑河被评为省级园林城市。安装雕塑小品等10组。规范治理市区商家牌匾127栋781户。出台了烟花爆竹燃放、架空管线管理等规范性文件5部。建立了城市精细化管理指标体系。全市新开工建筑面积223万平方米，其中市区65万平方米。改造棚户区71.5万平方米，落实实物配租房800套，改造农村泥草房7200户。

——把强化公共服务作为构建和谐黑河的重要任务，着力保障和改善民生。城镇新增就业2万人，登记失业率3.6%。安排见习毕业生1006名，招募“三支一扶”62人。市直提高了机关事业单位津补贴标准，人均补贴额度处于全省中上游水平。城镇居民基本医疗保险新增参保8万人。市直职工基本医疗保险住院费最高支付限额由2万元提高到3万元，六种大病门诊医疗费纳入统筹基金支付范围，新农合财政补助标准由人均80元提高到120元。全省首批新农保试点爱辉区参保农民2.8万人，参保率87%。为7075名老年人发放了交通补贴，市第二福利院投入使用，儿童福利院和流浪未成年人救助保护中心主体完工。高中阶段毛入学率88.1%，建成12所义务教育标准化学校。城镇社区卫生服务机构发展到70家，覆盖面100%。市中医院主体工程完工。黑河被确定为全国人口计生综合改革示范市和“婚育新风进万家”活动示范市，人口自然增长率2.71‰。35个乡镇综合文化站通过国家验收。市新建广电中心投入使用，城区有线电视数字化整体转换完成。热线电话实现整合，群众诉求调处中心投入使用，进京访、到省访分别下降39.2%和15.3%。“文明过马路、礼让斑马线”活动深入开展，交通秩序明显好转，文明礼让蔚然成风。安全生产形势总体稳定。刑事案件发案量下降1.7%，现案破案率提高2.5个百分点。边境管理进一步加强，边境秩序持续稳定。老区建设得到加强。民族宗教、地震、侨务、妇女儿童、残疾人、档案、地方史志等工作取得新成绩。

——把增强执行力作为加强政府自身建设的有效手段，着力提高行政效能。主动接受人大依法监督和政协民主监督，125件建议和提案全部办结。继续实施“十项绩效工程”，开展 “五个一”活动，促进了工作落实。落实民情恳谈制度，召开恳谈会48次，解决涉及群众切身利益的问题331项。加强公务员管理，新批准的参照公务员管理单位全部纳入绩效考核范围。制定了突发事件总体应急预案和49个专项预案。出台了重大决策社会稳定风险评估方案，共审核重大决策20项。政务公开扎实推进，重大决策、政府采购等信息在政府网站发布，政务查询系统加挂了俄文模块，开展了行政事业性收费政务公开试点。制定了政府系统领导班子及工作人员廉洁从政若干规定，党风廉政建设得到加强。

各位代表，2010年全市经济社会发展各项目标顺利实现， “十一五”规划任务圆满完成。这是省委、省政府和市委正确领导的结果，是市人大依法监督、市政协民主监督、社会各界大力支持的结果，是全市人民坚定发展信念、团结奋斗、顽强拼搏的结果，也是中省直各部门维护大局、积极参与的结果。特别是金融部门拓展经营领域，扩大信贷规模；电力部门强化基础建设，满足电力需求；石油部门积极争取指标，保证油料供应；工商部门主动帮企解困，提供优质服务；电信部门完善通讯网络，畅通信息渠道；口岸联检部门创新服务模式，提高通关效率；驻黑部队官兵牢记军人使命，维护边境安宁，等等。在此，我代表市政府，向辛勤工作在各行各业的全市人民，向给予政府工作大力支持的各级人大代表、政协委员和各民主党派、工商联、无党派人士、人民团体，向关心支持地方工作的驻黑中省直单位和人民解放军、武警官兵，表示崇高敬意和衷心感谢！

在充分肯定成绩的同时，我们更应看清前进中的困难和问题。主要表现在：经济总量小、发展速度不快仍然是我们面临的最大问题。产业结构不合理，产业项目比重小，缺少立市立县大项目；农业基础设施薄弱，产业化经营水平较低，县域经济发展不平衡，城镇化率不高；工业经济拉动不强，新兴产业发育缓慢，原字号、初加工等传统产业比重大；对外贸易结构不优，受政策因素影响大。社会管理有待加强，社会事业还有欠账，基本公共服务体系还不完善，有些干部的工作作风还不够扎实，等等。这些问题，我们将在今后工作中深入研究，认真解决。

二、“十二五”时期前景展望

“十二五”时期是我市全面建设小康社会的战略机遇期，也是乘势而上、加快发展的关键期。一方面，国家坚持扩大内需战略，推进发展方式转变，将出台一系列扶持政策。新增千亿斤粮食产能工程加快推进，大小兴安岭生态保护与经济转型启动，中国东北与俄远东地区合作步入机制化轨道，绥满沿边开放带有望上升为国家战略。我省继续推进“八大经济区”、“十大工程”建设，规划了“十大产业”，全省总体发展思路和格局基本确定，为我市实现跨越式发展创造了良好的外部条件。另一方面，我国今年通胀预期明显增强，将进一步紧缩信贷规模，宏观调控政策更加复杂多变。省内各地市发展热情空前高涨，竞争更加激烈，我们既面临与先进地区进一步扩大差距的压力，也面临节能减排、建设资金和技能人才等发展要素的制约。面对外部环境的不断变化，我们唯有抢抓机遇、自我加压、攻坚克难，才能尽快迎头赶上，才能绘就未来五年宏伟蓝图，才能实现强市富民目标。

今后五年，我们要根据市委四届十四次全会的要求，坚持以邓小平理论和“三个代表”重要思想为指导，深入贯彻落实科学发展观，以满足人民过上更好生活的新期盼为根本出发点，以推进全市更好更快更大发展为主题，以改革开放和科技进步为动力，以产业发育和转变经济发展方式为主线，以大项目和人才建设为支撑，以现代农业、“三新”产业、矿业经济和旅游、贸易为重点，实施投资与消费并重、开发与开放并举、保护与发展双赢、城乡和区域一体发展的战略，提高人民生活水平，维护社会和谐稳定，推动全市经济社会实现新的跨越式发展，为全面建成小康社会奠定更加牢固的基础。今后五年的主要预期目标是：到2015年，全市生产总值比2010年翻一番，年均增长13%以上，人均生产总值达到4400美元。全社会固定资产投资年均增长30%。社会消费品零售总额年均增长15%。全口径财政收入年均增长22%，四年翻一番。对外贸易进出口总值年均增长15%。城镇居民人均可支配收入和农村居民人均纯收入均年均增长12%以上。万元地区生产总值能耗年均下降3%。

为实现以上目标，我们要突出一个主题：加快发展，这是政府工作的首要任务。把握一条主线：加速转型，这是科学发展的本质要求。做好两个统筹：城乡统筹和区域统筹，这是实现全市协调发展的重要途径。今后五年，我们将全力推进五个行动区建设，以十项绩效工程为载体，把实现长期发展目标与完成阶段性任务紧密结合起来，认真抓好工作落实，努力在全省发展大局中找位置、求突破。这是全市“十二五”时期的总体战略安排。我们坚信，黑河一定能够成为体制机制更加完善、经济结构更加优化、生态环境更加良好、社会建设更加和谐、发展势头更加强劲的重要经济增长区域，一定能够为全市人民创造更多的福祉！

三、2011年工作目标和主要任务

做好今年工作对“十二五”发展具有重要意义。我们要认真贯彻市委四届十五次全会暨经济工作会议精神，突出上项目、育产业、健体系、成规模发展主题，强化综合开发、全面开放、深化改革、促进和谐各项措施，努力提高经济发展水平、城乡建设水平和改善民生水平，为“十二五”时期实现新的跨越式发展奠定良好开局。主要预期目标是，全市生产总值增长13%。全社会固定资产投资增长30%。社会消费品零售总额增长15%。全口径财政收入增长20%。对外贸易进出口总值24亿美元。城镇居民人均可支配收入和农村居民人均纯收入均增长12%。万元地区生产总值能耗下降3%。着重抓好以下工作：

（一）开展“产业项目建设年”活动，增强经济发展后劲。紧紧围绕我市确定突出发展的“十大产业”，将产业项目作为推动发展的强力支撑，加大推进力度。开工和建成投产重点项目88项，年度投资185亿元，其中产业项目54项，年度投资67.1亿元。全力推进重点产业项目建设。加快嫩江大多宝山铜矿、逊克翠宏山铁多金属矿、爱辉争光岩金矿、合作区多晶硅和聚乙烯醇、爱辉燕麦加工和酒类酿造储藏一体化、北安天华风电、嫩江鲜奶加工、爱辉龙源风电和金湾水电站、逊克乌二水电站等项目建设，确保完成年度投资计划。抓好爱辉金三角煤矿扩能改造、逊克东安岩金矿、合作区维生素C和聚乙烯管材、爱辉林板一体化、五大连池啤酒、嫩江生猪加工和海信热电、北安汉麻纺织和整体浇铸式节能墙体等项目推进，力争年内建成投产。做好土地、资金、建材等各项准备，爱辉同兴煤矿、生物制浆、太阳能并网发电、水飞蓟产业化和生物制药产业基地、北安风电设备制造等项目开工建设。做好项目前期工作。围绕国家和省投资导向，谋划一批科技含量高、市场竞争力强的项目，做深做细做实各类报件，加快推进27项重点储备项目。抓紧完善合作区多晶硅切片、嫩江生物质发电、五大连池和逊克风电等项目前期。完成合作区油品储运、嫩江嵩天小麦加工等项目开工前全部工作，力争开工建设。继续加大招商引资力度。主动拜访国内外500强企业和大专院校，争取引进更多有一定科技含量的企业。坚定不移地抓好驻外招商组工作，市级各组力争全面完成既定任务，各县（市、区）也要全面推行这一做法，下指标、定任务、落责任，动员更多的人参与招商引资，到位资金增长20%以上。

（二）推进局市合作共建，促进现代农业发展。继续实施“双增”工程，促进农业发展和农村建设，加快城乡一体化进程。农业增加值增长4%以上。粮食总产稳定在230万吨以上。抓好农业生产。调整种植结构，大豆面积调减10%，玉米、小麦、水稻、马铃薯面积均增长10%。绿色有机农产品种植面积850万亩。土地流转面积增长10%，规模经营面积增长20%。大力发展林业经济，完成省预安排造林计划32万亩，开展森林培育15万亩，北药种植22万亩，林业产业产值增长11%。完成县级林地保护利用规划编制，保护湿地资源，力争新增省级湿地保护区2处。加快畜牧养殖“三带三区”建设，牧业增加值增长10%。建设江鱼和矿泉鱼养殖基地，渔业产值增长15%。对俄土地开发达到95万亩，增长5.6%。转移农村劳动力17.3万人，实现劳务收入11.3亿元。强化基础建设。实施农田水利、农业机械、培肥地力、人工影响天气四项基础工程。建设北安和嫩江节水灌溉工程，增加灌溉面积12万亩。新建农村饮水安全工程，解决40个村屯饮水问题。完成黑河城区堤防、孙吴和逊克护岸7.5公里。实施农机购置补贴项目，地方续建新建现代农机合作社77个，完成整地1000万亩。借助农场优势，探索共建农机合作社，完成“三代”面积700万亩。实施测土施肥1000万亩。局市联建农业示范园区25个，推广模式化栽培550万亩。整建人工影响天气作业点，建设黑河气象雷达基地和飞机增雨基地。提高产业化水平。扶持北大荒丰缘麦业、九三油脂及北安恒泰薯业、嫩江翔河油脂、五大连池开发麦业、逊克茂田牧业、孙吴沙棘、爱辉山珍等企业扩能壮大，着力引进大豆、玉米、北药等精深加工企业，推进九三、北安、嫩江、五大连池等农产品加工园区建设，全市农产品加工龙头企业达到60户，加工能力达到500万吨。推进农产品基地建设，完善加工企业、经济组织与农户间利益联结机制，全市农村合作经济组织达到700个，加入农户超过50%。推进城乡一体化示范镇建设。培育主导产业，罕达汽、多宝山镇矿业产值比重达到50%以上，爱辉、五大连池镇旅游业产值比重达到23%和48%以上，赵光、通北、双山和龙镇工贸物流业产值比重达到35%以上，辰清、逊河镇农牧业产值比重达到45%以上。加快人口扩张，低于2万人的示范镇人口增幅30%，高于2万人的增幅15%。局市共建双山示范镇，完成双吉小区一期工程，启动二期工程和商贸市场、给排水管网等项目。合作推进风景区旅游名镇建设。加快6个 新型社区及100个新农村重点村建设，积极推进沿黑龙江、沿202国道、沿齐嫩高速新农村示范带建设。开展村容整洁行动。搞好村屯硬化、绿化和净化，规范房屋、庭院、边沟、厕所和垃圾场，每个县（市、区）30%以上乡镇、每个乡镇30%以上村屯、每个村屯30%以上农户达标。市级文明村至少有一条主干街路实现硬化亮化，街路两侧设置定点垃圾箱。

（三）创造良好发展条件，促进工业经济快速壮大。支持和服务好工业经济发展，规模以上工业增加值增长15%以上，地方规模以上工业利税增长12%以上。提高园区承载能力。进一步完善五秀山、二公河俄电加工区基础设施，协调省电力公司加快输变电设施建设，保证园区电力供应。推进爱辉对俄进出口加工基地道路、供电、给排水等工程，增强综合服务功能。加快北安新能源战略产业园、嫩江及其他工业园区的基础配套，抓好园区环境建设，满足项目入区条件。完成边境经济合作区区域置换核准工作。加强矿产资源勘查。认真落实矿产资源勘查开发管理决定和实施意见，严厉打击违法违规行为，确保矿山企业规范健康发展。发挥盛源公司平台作用，加强与国内大型矿业企业合作，力争取得实质性进展。利用小兴安岭地区1∶5万航空物探测量成果，推进嫩江多宝山、逊克宝山等重点区域矿产详查，为上新项目创造基础条件。矿产品质量监督检验中心开工建设。抓好节能减排工作。推进电力、冶金、建材、工业硅等重点行业节能降耗，核定企业主要污染物总量控制指标，分解落实任务。淘汰落后产能，关闭能耗高、污染严重的小企业。加快煤炭、铜、金等矿山节能减排和环境治理，抓好矿山环境治理保证金收缴。鼓励企业利用新技术改造设备，提高产品附加值，降低单位产值能耗，实现资源利用最大化。同时，加强工业运行监控，组织好煤电油运供需衔接，帮助企业解决实际困难。加快交通路网建设。强化省管项目服务，前嫩公路北安至嫩江段143公里完成路面工程、北安至风景区42公里高速路交工通车、101公里二级路完成桥涵和路基工程，吉黑高速北安至黑河段242公里以及北黑辅道219.5公里、绥北高速北安段79公里、市区外环路11.2公里交工通车。保证市管项目建设进度，加黑公路黑宝山至罕达汽段完成路面升级改造83公里，嫩黑公路215.4公里全线路基贯通。北五铁路开工建设。

（四）放大内外互动效应，提升对外开放质量和水平。加强地区间合作，坚持外经贸量的扩张与质的提升并重，增强对地方经济的拉动作用。努力增加本港进出口额。抓住中俄两国能源合作机遇，落实边境贸易进口扶持政策，合作开发远东地区煤炭等矿产资源和化工原料，对俄进口额增长10%。扩大服装、农副产品、工程机械、农用机械等出口，吸引企业开展进口加工。水泥、蔬菜、工业硅等地产品出口额增长15%。完善北安、爱辉果蔬出口基地加工仓储功能，形成对俄果蔬出口产业链。加快民间贸易方式转型，规范运作对俄旅游自带货物出口，不断扩大出口份额。深化对俄投资合作。引导企业“走出去”，加快合作项目建设。水泥粉磨站完成设备安装调试，建筑装饰材料综合市场和赤塔重汽二级服务站投入运营，布市重汽4S店二期加快建设。推进东方明珠煤炭开采项目，增加煤炭开采量。进一步完善互贸区功能，强化管理和服务。搭建交流平台。完善与阿州布市会谈会晤机制，落实好年度政府间会谈纪要。加强与涅留恩格里、伊尔库茨克、雅库茨克、后贝加尔等地沟通联络，主动与俄腹地其他城市建立密切联系。加强企业间和各界交往，提高教育、文化、卫生、环境保护、安全应急等领域合作水平。继续举办大黑河岛国际经贸洽谈会。提高口岸输运能力。探索航运管理改革，发挥口岸平台作用。推行集装箱运输，加快口岸货物周转。浮箱固冰通道投入使用。推进货检二期、旅检码头建设。继续推进黑龙江大桥前期工作。

（五）拓展服务消费领域，增强消费拉动作用。强化旅游龙头地位，改善消费环境，加强策划宣传，聚集人气商气。接待旅游者312万人次，增长20%，实现收入43亿元。壮大“四型经济”。全力组织攻关，力争申遗成功。加快五大连池旅游名镇建设，新建环湖景观道路和新期火山观光栈道，启动火山博物馆、SPA城和公共设施等项目，加快基础设施建设。五大连池机场力争开工。格球山景区对外开放。继续举办火山圣水节。加快其他景区建设，中俄民族风情园新建达斡尔风情区，大黑河岛游乐园投入运营，孙吴胜山旅游区完成广场建设，瑷珲仿古街改造启动临街建筑立面装饰。新建西沟试车场和孙吴试车综合楼等项目，试车收入增长15%。组织承办各级各类会议及大型会展等活动，聚集人员增长10%。培育新兴消费产业。出台优惠政策，开发特色住宅，吸引国内及俄公民来购房居住和旅游购物，壮大商住消费群体。扩大黑河学院、黑河一中招生规模，促进教育消费。办好特色医院，吸引俄罗斯人就医。支持金融部门开展购车贷款业务，鼓励居民购车消费。加快黑河、北安、孙吴物流中心和嫩江物流园区建设，带动新兴服务消费。改善消费环境。加快高档次接待设施建设，黑河龙江国际交流中心完成主体工程，五大连池国际旅游接待中心投入使用。风景区开工建设四星级以上酒店2家、高档疗养院3家。完善重点景区旅游标识及购物、娱乐等配套设施，城市大型商场和重点景区全部实行中俄双语明码标价。协调旅检部门延长服务时间，加快通关速度。继续推进“万村千乡”市场工程，落实家电以旧换新等促销政策。加强市场监管，提高投诉案件处理效率，打击非法导购、哄抬物价等行为。

（六）壮大财政实力，提高财税金融保障能力。广开财源，优化支出，提高资金使用效益，支持和保障经济社会发展。努力增加财税收入。争取合作区列入首批省级财源建设示范园区，油品储运等5个项目纳入省重点财源扶持范围。进一步完善煤炭业税费征管机制，堵塞漏洞。完善耕地占用税征管机制，扩大土地使用税征收范围。加强建筑施工现场营运车辆和租赁机械设备税收管理。准确核定饮食、洗浴、娱乐场所税赋定额。启动市区初始房屋登记契税征收。对水泥、石灰石企业和铜矿征收价格调节基金。对房地产和建筑业新建项目实行电子软件管理，扩大非税收入电子化票据改革实施范围。提高工商税收占一般预算收入比重、一般预算收入占财政收入比重、财政收入占生产总值比重。保证重点支出。出台财政支出项目绩效预期目标管理办法，开展精细化管理年活动。规范部门预算管理，从紧安排一般性支出和追加项目支出。逐步完善公共财政体系，确保民生保障支出逐年增长。深化国库集中支付改革，市直全面推行公务卡消费，扩大政府采购范围，启动财税库银联网工作。提高向上争取针对性。深入研究国家和省产业政策导向，把目标分解到每个战线和部门。建立市级领导、相关部门、各招商组和各县（市、区）政策信息共享机制。向上争取资金增长10%以上。鼓励金融机构支持地方发展。完善政银企平台和信用体系建设，落实金融机构支持地方经济发展考核奖励办法，扭转存贷差过大局面。推进农村信用社产权制度改革，加快组建农村商业银行。金融机构为企业投放贷款额度平均增长15%以上。

（七）加强规划建设管理，打造宜居宜业宜乐的幸福城市。扎实推进“三优”文明城市创建工程，塑造特色鲜明的城市形象。做好规划设计工作。扩大规划覆盖面，年度新开发地段全部完成修详规划。严把建筑风格、造型、结构、色彩关。严把高层建筑消防设计关，严格验收标准。完成市区测绘及地形图制作项目。做好县（市）城市总体规划修编和示范乡镇规划编制工作。强化建设项目批后管理，实行半月报备制，建立检查档案，严肃查处违法建筑行为。推进市政重点工程建设。市区新建道路2条，改造27条，立面改造装饰楼体100栋。改造小区47个。改造7条街路牌匾1500块。建设东南部公园，实施黑龙江公园东扩工程，增加绿地30万平方米。启动创建国家级园林城市，力争3年完成。增设雕塑、候车亭等室外家具。推进机场路出口两侧、西部棚户区改造工程。建设黑河城市规划展览馆，与黑河学院共建体育馆。启动创建国家级环保模范城，完成市区污水处理厂二期和北安垃圾处理、逊克污水处理工程，开工五大连池双泉水源地污染防治综合工程，推进北安滨水公园二期和孙吴滨水公园三期，加快嫩江滨水城市建设。提高城市建设和管理标准。建立健全市县两级城市建设指标体系。提高道路建设标准，按照城市发展布局和人车流量合理规划路网，抓好“两覆盖一平整”工程。提高楼房建设和立面改造标准，加强专家评审力度，使主要建筑都成为精品，主要街路都成为景观。提高环境卫生管理标准，将环卫作业细化到路段和责任区。提高绿化建设标准，扩大公园面积，提高城区绿化水平。市区及各县（市）开展乱搭乱建、空中拉线、乱贴乱画等8个方面专项整治。完成数字化城管平台基础建设和基本数据采集录入。爱辉、逊克、孙吴要利用好省边境城市建设专项资金，抓好主街路建筑立面改造工作。其他县（市）也要加大投入，做好城市建设和小区改造工作，全面推行“五化”管理，“三优”文明城市创建工程要走在全省前列。

（八）统筹发展各项社会事业，提高公共服务水平。坚持以人为本，努力保障和改善民生，推动社会全面进步。强化科技和人才培养引进工作。继续实施《全民科学素质行动计划纲要》，不断提高科学素质。建立中俄高新技术引进与合作机制，创建国家级TRIZ理论基地。组建行业人才协会，建立新兴产业领域学科梯队。完善人才优惠政策，本地人才在专业领域取得与外来人才相同业绩，享受同等待遇。为重点企业、大项目引进培养技能人才400人，引进教育、医疗、规划和矿产等行业领军人才和紧缺高层次人才50人，引进国外人才50人，申报引智项目14个。输送本地专业技术人才外出培训130人。促进教育事业发展。加大学前教育投入力度，扩大幼儿园所覆盖面。提高教学质量，减少生源流失，小学和初中辍学率分别控制在1%和3%以下，高中阶段毛入学率达到90%以上。开工建设职教中心教学实训综合楼，做好高职学院申报准备工作。深化职业教育改革，培养高技能人才500人，培训农村实用人才和新型农民2万人次。完善3所特殊教育学校设施。深化中俄青少年交流合作。提高医疗服务水平。深化医药卫生体制改革，做好省级公立医院改革试点工作，推进基本医疗和公共卫生服务保障制度建设，完善基本药物制度。市中医院、孙吴医院、五大连池医院二期投入使用。加大政府对公立医院的投入，市第一医院争创三级乙等医院。巩固新农合成果，报销额度封顶线达到当地农民人均纯收入6倍以上。垦地互设医保定点单位，统一收费标准。加大食品药品监督力度，做好重大传染病预防控制工作。继续巩固低生育水平，人口自然增长率控制在5.3‰以内。市区启动国家级卫生城市创建工作。繁荣文化体育事业。举办第二届中俄文化大集、第三届“金色旋律”农民艺术节。建设乡镇综合文化站5个、 “农家书屋”100个。做好国家重点文物保护单位及省级文化生态保护园区申报工作，建设非物质文化遗产鄂伦春族、满族、达斡尔族传承传习基地。推进黑河生态地质博物馆建设。成立中远国际传媒公司，探索以股份制模式推进文化产业。发展体育事业，提高竞技水平，广泛开展全民健身运动。同时，进一步做好老区、民族宗教、地震、侨务、妇女儿童、老龄、人防、档案、地方史志等工作。

（九）保障和改善民生，营造和谐稳定社会环境。在促进发展的同时，关注城乡弱势群体，让广大群众共享发展成果。提高城乡居民收入。认真落实收入分配改革政策。适当提高市直公务员和事业单位人员津贴标准。严格执行最低工资制度，企业根据经营状况，适度增加职工工资收入。增加农民工资性收入。根据市场波动，适时加大价调基金投放力度。扩大地产蔬菜储备，保持蔬菜价格稳定。做好就业再就业工作。城镇新增就业1.6万人，登记失业率控制在4.5%以内。落实优惠政策，安排小额担保贷款1亿元。建立创业指导服务中心，培训创业人员4000人，职业介绍2.3万人。新增园区项目建设用工岗位1万个，开发公益性岗位1000个，零就业家庭至少1人实现就业。完善社会保障体系。城镇居民基本医疗保险、新型农村养老保险和失业保险参保面分别达到90%、35%和60%。制定居民门诊医疗费用社会统筹办法。市直职工医疗保险最高报销额度提高到4万元，医保个人账户单位缴费部分划转比例提高0.2～0.4个百分点。居民住院医疗费报销比例提高到60%以上。及时调整失业金、工伤津贴和养老金标准。启动第二批新型农村养老保险试点，参保人数9.5万人。对考入大学的残疾考生、残疾人家庭的考生给予补助，发放残疾人交通补贴。完善“九制一体”社会救助体系，社会救助率达到97%以上。儿童福利院和流浪未成年人救助保护中心投入使用。推进城市和谐社区建设，扩大农村社区覆盖面。保持社会安全稳定。深入开展安全隐患排查和重点行业领域专项整治，坚决遏制重特大事故，努力控制事故总量。创新和完善信访工作机制，运用综合手段化解矛盾，最大限度维护群众利益。深化森林防火“两个机制”和“五个系统”建设，维护生态安全，保护森林资源，确保不发生重特大森林火灾。建立现代警务指挥体系、社会治安防控网络、打防管控一体化运作机制，继续实施“警灯闪烁”工程，推进公安信息化“金盾工程”二期建设，深入开展“文明过马路、礼让斑马线”活动。创新流动人口服务管理、特殊人群帮教管理、网络虚拟社会建设管理，以及新经济组织、新社会组织规范管理。全面启动“六五”普法工作。加强国防教育，争创国家级双拥模范城“两连冠”。深化边防“三个体系、三个机制”建设，强化边境管理，确保边境安定。

各位代表，我们在统筹兼顾的同时，突出保障和改善民生，今年要为群众办好十件实事。 一是安排见习、“三支一扶”和企事业单位就业岗位2700个。二是发放“三无”人员和低保人群物价补贴。三是扩大社会保险覆盖范围，启动事业单位工伤保险。四是对关停并转企业、改制后没有管理主体和有管理主体但经营亏损的企业退休人员给予供热费补贴。五是新建标准化学校8所，改造D级危房9.66万平方米。六是按照人均不低于15元的标准，落实基本公共卫生服务经费。加大对公立医院基本检查设备的投入。七是增加市区群众活动中心数量。八是建设300套公租房和2225套廉租房。九是改造建设农民住宅9700户62万平方米。十是完善城市供热系统，市区增加供热面积60万平方米。

四、加强政府自身建设

以公众意志和利益为核心，转变职能，加大力度，依法管理社会事务，不断提高公共服务水平，为实现跨越式发展提供坚强保障。建设学习型政府，增强跨越发展的坚定性。用科学发展观武装头脑，完善领导干部和公务员学习制度，增加知识储备，提高理论水平，创新发展理念。认真研究利用好国家、省政策，紧密联系我市实际，深入基层开展调研，增强分析问题、研究对策、解决矛盾的能力。建设服务型政府，增强亲民为民的主动性。坚持民情恳谈制度，落实领导干部接待群众和工作联系点制度，完善便民利民措施，帮助广大群众尤其是困难群体解决生产生活难题。全面推进政务公开，提高政府工作透明度。加强电子政务建设，支持鼓励网络问政，畅通政府与群众的沟通渠道。健全突发公共事件应急机制，提高快速反应能力。建设法治型政府，增强施政行为的规范性。自觉接受市委领导，主动向人大报告工作，向政协通报情况，接受监督，及时办理议案提案。加强与民主党派、工商联、无党派人士和群团组织沟通，主动接受社会公众和新闻舆论监督。严格依法行政，进一步规范执法行为。健全政府重大事项决策公示制度、听证制度和专家咨询制度，提高科学决策、民主决策水平。进一步完善权力运行机制，简化审批手续，提高行政效率。建设效能型政府，增强执行落实的实效性。继续实施“十项绩效工程”，开展“五个一”活动，量化目标任务，明确责任主体，完善考核办法。加大督办落实力度，强化跟踪问效，切实提高执行力。严格执行行政问责制度，坚持有错必究。精简规范会议和文件，腾出更多的时间和精力谋发展、抓落实。建设廉洁型政府，增强拒腐防变的自觉性。加强惩防体系建设，认真落实党风廉政建设责任制，严格执行廉洁自律各项规定。加强领导干部经济责任审计和政府重大投资项目管理。强化工程建设、产权交易、土地管理等重点领域的政府层级监督和专门监督，加强对人财物的管理，严肃查处各类违法违纪案件。

各位代表，后发赶超的历史重任呼唤我们奋发有为，竞相发展的激烈态势昭示我们不进则退。让我们在市委的坚强领导下，紧紧依靠全市人民，以百倍的信心和干劲，为实现“十二五”开局之年的各项目标，为经济和社会的更好更快更大发展，为人民群众更加富裕而努力奋斗！
